# A Case of Nodal Marginal Zone Lymphoma Presenting as Left Leg Swelling

**DOI:** 10.7759/cureus.83013

**Published:** 2025-04-25

**Authors:** Megana Ballal

**Affiliations:** 1 Department of Medicine, University of California, Los Angeles (UCLA), Los Angeles, USA

**Keywords:** b-cell neoplasm, marginal zone lymphoma, marginal zone lymphoma subtypes, nodal marginal zone lymphoma, nodal mzl

## Abstract

Nodal marginal zone lymphoma (NMZL), a rare subtype of non-Hodgkin lymphoma, typically presents with widespread lymphadenopathy, often without constitutional symptoms or extranodal involvement. Here is a case of a 63-year-old woman who presented with left leg swelling and was found to have bulky lymphadenopathy on imaging. After an initial lymph node biopsy revealed an alternate diagnosis, a repeat biopsy was pursued, given the high suspicion for malignancy. Given her indolent disease course and lack of significant symptoms, the patient opted to defer systemic therapy in favor of active surveillance with close monitoring. This case highlights the challenges in the diagnosis of NMZL.

## Introduction

Follicular lymphoma and marginal zone lymphoma (MZL) are both types of non-Hodgkin lymphoma (NHL) and are considered indolent mature B-cell neoplasms [1]. MZL is the second most common indolent lymphoma and represents 7% of NHLs [2]. There are three MZL subtypes: extranodal MZL of mucosa-associated lymphoid tissue (MALT lymphoma), splenic MZL, and nodal MZL (NMZL). MALT lymphoma comprises 70% of cases, followed by splenic MZL (20% of cases) and NMZL (10% of cases) [2]. Notably, there is geographic variation regarding the prevalence of NMZL. A review of data from the United States Surveillance, Epidemiology, and End Results (SEER) 18 program, which is a collection of cancer registries, showed that 30% of MZL cases were nodal [3]. While there is some overlap with phenotypic features, these subtypes differ in terms of their predisposing conditions and clinical presentation [1].

## Case presentation

A 63-year-old woman presented to the emergency department with left leg swelling. Four months earlier, she had presented with similar symptoms; a lower extremity ultrasound at that time was negative for deep vein thrombosis, and she was discharged with primary care follow-up and instructed to elevate her extremity. Four months after that initial presentation, she noticed that the swelling had increased, so she came for further evaluation. 

The initial review of systems for the patient was negative. Her past medical history included diabetes mellitus, for which she took metformin. She had no significant past surgical history. She was not a smoker and had no significant alcohol or illicit drug use. Vital signs on admission were unremarkable. Physical examination revealed 1+ pitting edema extending to the left thigh. Admission hemoglobin, creatinine, lactate dehydrogenase, and serum albumin are noted in Table 1.

**Table 1:** Laboratory test results CEA: carcinoembryonic antigen; CA: cancer antigen; SPEP: serum protein electrophoresis; IFE: immunofixation electrophoresis; IgG: immunoglobulin G

Parameters	Patient Values	Reference Range and Unit
Hemoglobin	10	11.6-15.2 g/dL
Creatinine	0.97	0.6-1.3 mg/dL
Lactate Dehydrogenase	315	125-256 U/L
Albumin	4.4	3.9-5.0 g/dL
CEA	1.5	<3.1 ng/mL
CA-125	14	0-35 U/mL
CA 19-9	12	0-35 U/mL
CA 15-3	14	0-35 U/mL
SPEP	No monoclonal bands	No monoclonal bands
IFE	No monoclonal immunoglobulins	No monoclonal immunoglobulins
IgG, serum	1990	700-1600 mg/dL

**Figure 1:**
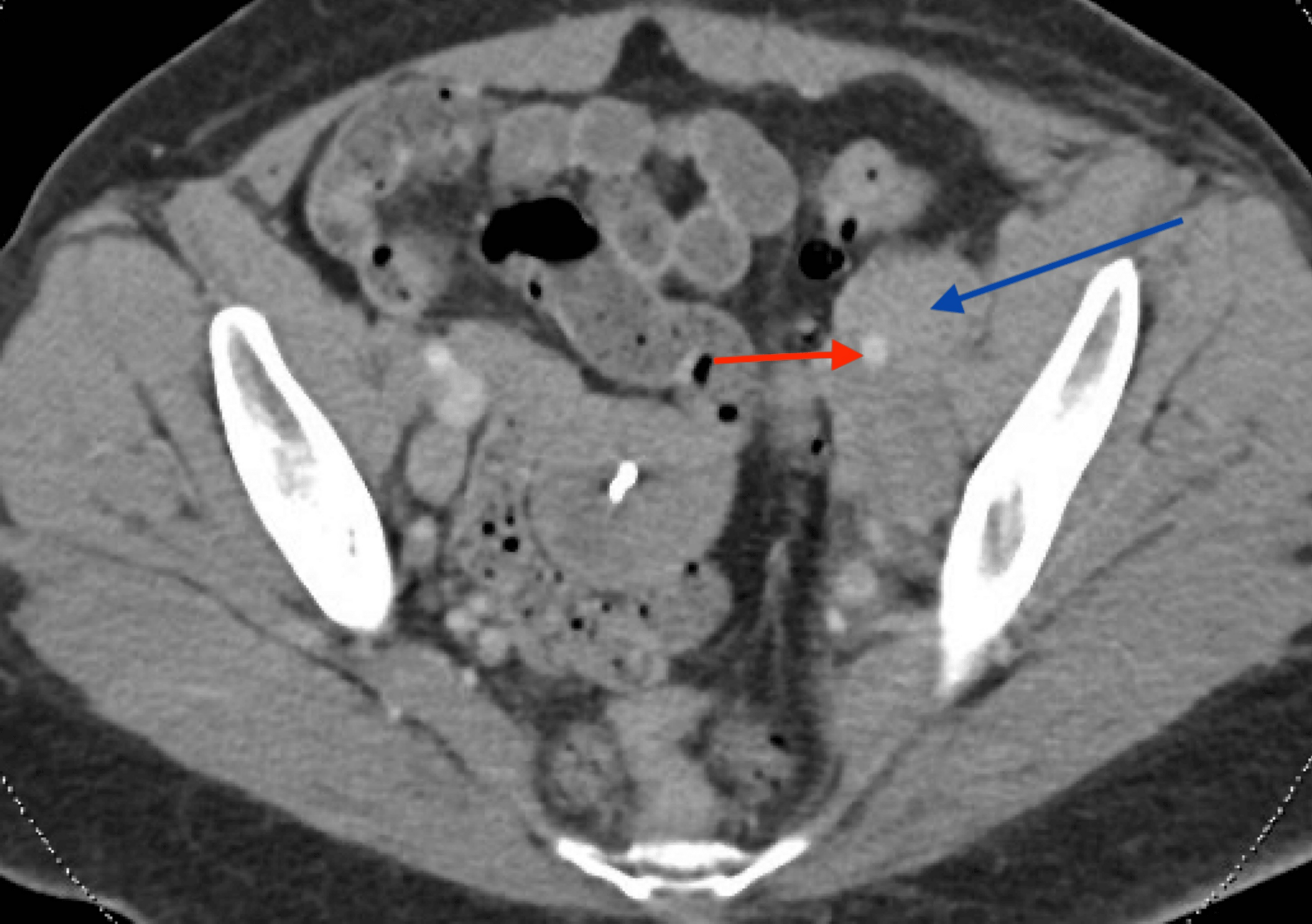
CT scan of the abdomen and pelvis Red arrow shows external iliac artery. Blue arrow shows confluent lymph nodes measuring 61x35 mm.

Urinalysis was negative for proteinuria. A prior screen for hepatitis C was also negative. Additional initial labs were otherwise unremarkable. Computed tomography (CT) scan of the abdomen and pelvis with contrast showed mild left hydronephrosis due to obstruction from lymphadenopathy, with findings concerning for superimposed pyelonephritis and pyelitis. Other significant findings included left greater than right-sided bulky confluent lymphadenopathy of the pelvic sidewalls, iliac, and retroperitoneal nodal stations (Figure 1).

**Figure 2:**
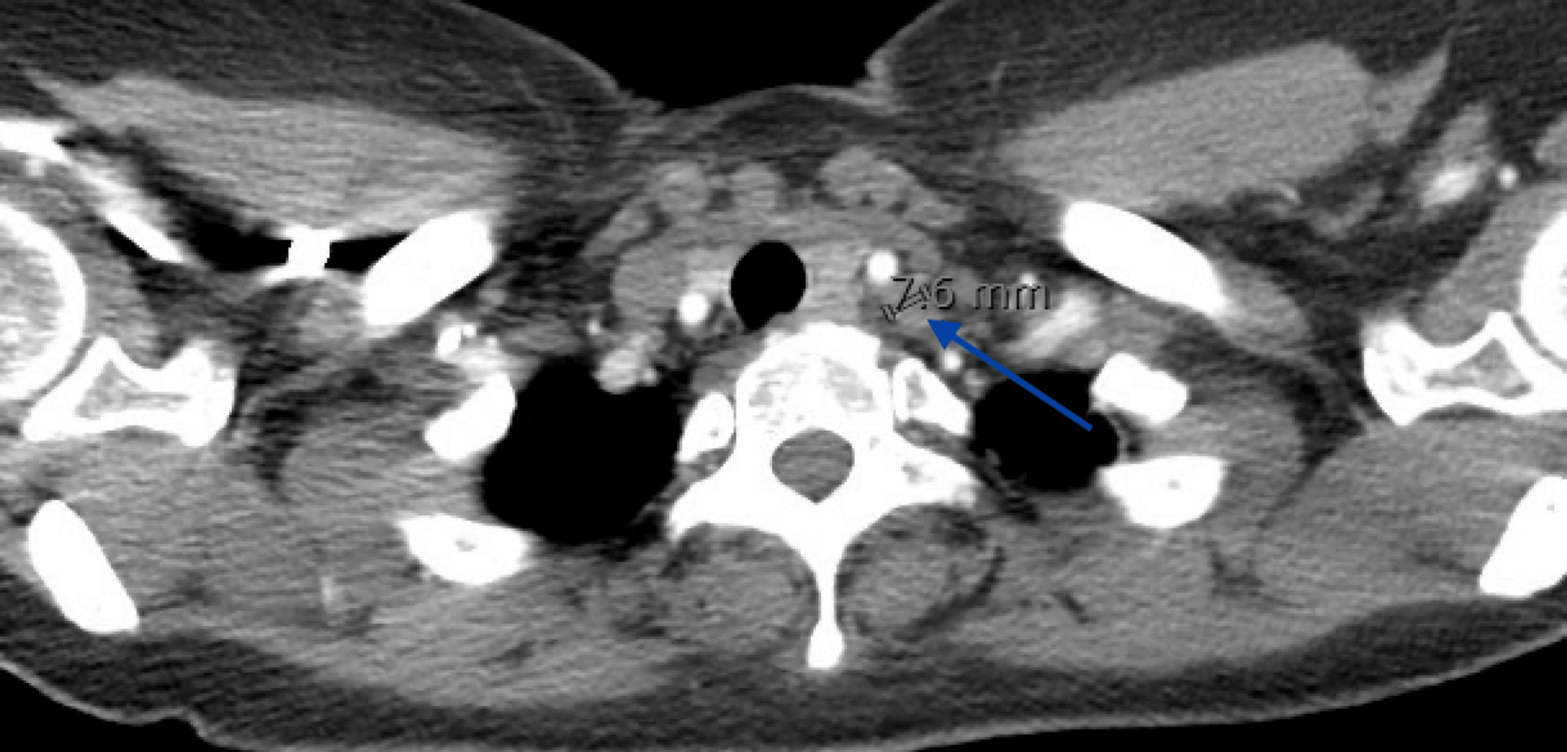
CT angiogram of the chest Blue arrow shows subcentimeter cervical lymph node

This was suspicious for malignancy, with etiologies including lymphoma or metastatic disease. There was also lymphadenopathy encasing and narrowing or occluding the left common iliac and left external iliac veins, as well as a right iliac bone sclerotic lesion. A CT angiogram of the chest was done to evaluate for malignancy and pulmonary embolism. Prominent subcentimeter left lower cervical and supraclavicular lymph nodes were noted on CT angiogram of the chest (Figure 2). 

The patient underwent a left inguinal lymph node biopsy. On further history, she reported possible urinary frequency. Given the CT findings and a urine culture with *Escherichia coli*, she was treated with a course of antibiotics. Given the imaging findings suggestive of malignancy, the patient was discharged with a referral to hematology-oncology for further diagnostic evaluation and management.

**Figure 3:**
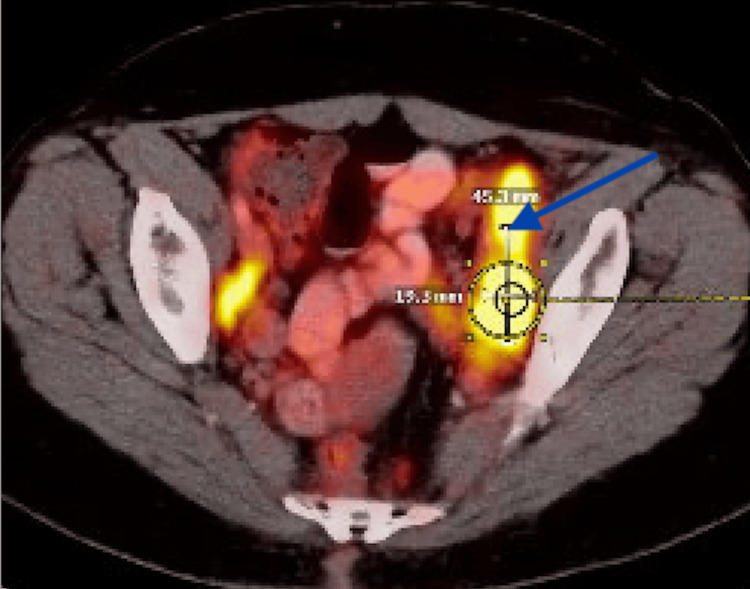
PET-CT scan Blue arrow shows intensely fludeoxyglucose-18 (FDG)-avid pelvic lymphadenopathy.

Initial lymph node biopsy suggested angiomyomatous hamartoma, a rare benign lesion, underscoring the diagnostic complexity and potential for initial misclassification in lymphoproliferative disorders. Concurrent flow cytometry did not detect a monotypic B-cell population. The patient followed up with hematology oncology and underwent additional blood tests, which are included in Table 1. Positron emission tomography (PET)-CT scan revealed intensely fludeoxyglucose-18 (FDG)-avid lymphadenopathy suspicious for malignancy (Figure 3).

**Figure 4:**
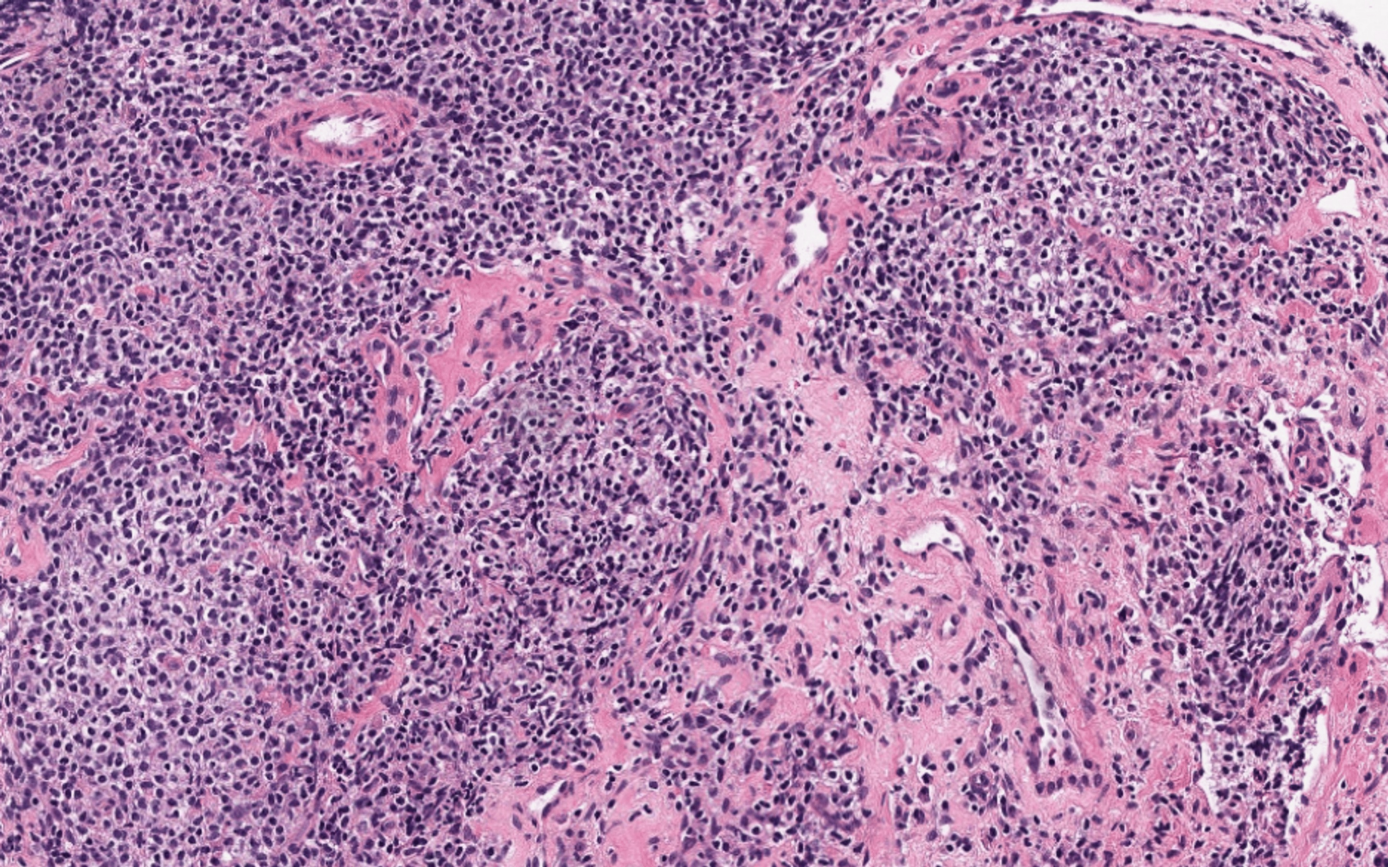
Section of lymphoid tissue containing small atypical lymphoid cells

**Figure 5:**
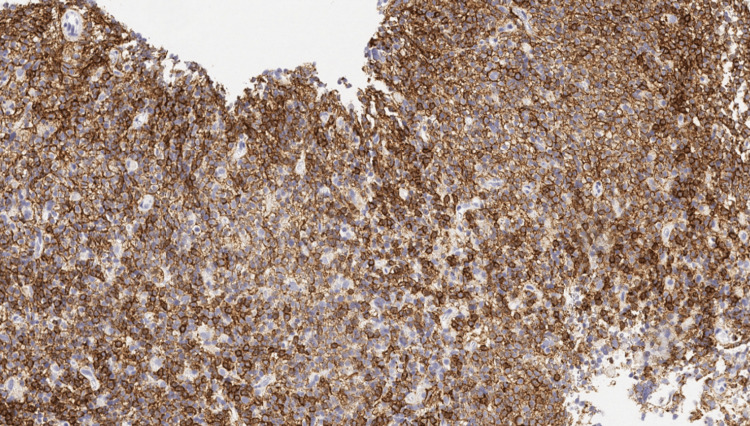
CD20 immunohistochemical stain

The patient subsequently underwent a left iliac lymph node biopsy. Histopathologic examination confirmed NMZL, revealing small atypical lymphoid cells with immunophenotyping positive for CD19, CD20, and CD23, and a low Ki-67 proliferation index, consistent with an indolent B-cell neoplasm (Figure 4, Figure 5).

A follow-up CT scan of the chest, abdomen, and pelvis two months later demonstrated stable lymphadenopathy. The patient remained asymptomatic with the exception of the compression on her veins as well as ureter related to bulky lymphadenopathy. The option of combination therapy with bendamustine and rituximab was discussed. She elected to defer treatment and preferred to monitor for worsening symptoms as well as continue surveillance imaging and labs.

## Discussion

NMZL is rare, making up less than 2% of NHLs [2]. It is defined as a B-cell neoplasm with lymphadenopathy and no splenic or extranodal involvement, with the diagnosis based on lymph node histology [4]. The median age at diagnosis is 60-70 years for all MZL subtypes [5]. The majority of patients present with disseminated nodal involvement [6]. A systematic review by van den Brand et al. showed that approximately 50% of patients present with stage III or IV disease, 10-20% have B symptoms, and generally, a minority of patients have anemia, thrombocytopenia, and peripheral blood or bone marrow involvement [7]. There are no unique chromosomal abnormalities consistently found with this MZL subtype [4]. NMZL has an association with hepatitis C infection [8]. Although there is a connection with chronic inflammation, there isn’t a clear association with autoimmunity, unlike with other MZL subtypes, and serum immunoglobulin M paraprotein is not often detected [6]. The detection of paraproteins may predispose patients to complications such as hyperviscosity syndrome, peripheral neuropathy, or renal dysfunction. The recommended imaging modality for staging and assessing treatment response is CT with contrast. The utility of PET/CT can vary based on MZL subtype, and the Lugano classification notes MZL to be a non-FDG-avid disease [5]. However, FDG-avidity is over 76% in patients with NMZL, so it is useful in select patients [5]. 

Of the MZL subtypes, extranodal MALT lymphoma is the best characterized, most common, and has the best prognosis [5]. While NMZL is generally regarded as incurable, its indolent nature often allows for a watch-and-wait approach in asymptomatic patients, even those presenting with advanced-stage disease [9]. Many patients live more than 10 years after their diagnosis [5]. Indications for treatment of NMZL include bulky disease, symptomatic lymphadenopathy, presence of B symptoms, and/or significant cytopenias [5]. 

The specifics of treatment depend on the patient’s characteristics. For example, given data showing lymphoma regression following treatment for hepatitis C, antiviral therapy is generally recommended in patients who don’t otherwise need immediate disease control [5]. Systemic therapy for symptomatic, advanced-stage NMZL typically involves rituximab in combination with chemotherapy, although rituximab may be offered alone if the patient is unable to tolerate chemotherapy. Examples of chemoimmunotherapy regimens include bendamustine-rituximab and cyclophosphamide-vincristine-prednisone-rituximab [5]. Treatment is individualized for those with relapsed MZL. For a small percentage of patients, NMZL can transform into a more aggressive lymphoma, such as diffuse large B-cell lymphoma, in which case management is also individualized [9]. Hematolymphoid malignancies often present with non-specific or atypical clinical features, posing significant diagnostic challenges, as exemplified by a case of spontaneous splenic rupture in chronic myeloid leukemia [10]. In the current case, isolated left leg swelling led to the diagnosis of NMZL, a rare indolent B-cell lymphoma subtype. These cases underscore the broad and sometimes deceptive spectrum of presentations in hematologic malignancies, reinforcing the importance of comprehensive clinical evaluation and awareness of rare manifestations.

## Conclusions

This case notes the complexities in diagnosing NMZL. This indolent B-cell neoplasm is rare, and the clinical presentation is variable, making the diagnosis more challenging. Treatment can vary based on the patient’s characteristics, with active surveillance being an option for asymptomatic patients even with advanced disease. Our understanding of NMZL has advanced over the past decade based on insights from clinical presentations as well as histological and molecular findings. Ongoing research on NMZL is focused on understanding its molecular characteristics and evaluating targeted therapies to improve treatment strategies.

## References

[1] Laurent C, Cook JR, Yoshino T, Quintanilla-Martinez L, Jaffe ES (2023). Follicular lymphoma and marginal zone lymphoma: how many diseases?. Virchows Arch.

[2] Cheah CY, Seymour JF (2023). Marginal zone lymphoma: 2023 update on diagnosis and management. Am J Hematol.

[3] Cerhan JR, Habermann TM (2021). Epidemiology of marginal zone lymphoma. Ann Lymphoma.

[4] Alderuccio JP, Kahl BS (2022). Current treatments in marginal zone lymphoma. Oncology (Williston Park).

[5] Peters A, Keating MM, Nikonova A, Doucette S, Prica A (2023). Management of marginal zone lymphoma: a Canadian perspective. Curr Oncol.

[6] Thieblemont C, Molina T, Davi F (2016). Optimizing therapy for nodal marginal zone lymphoma. Blood.

[7] van den Brand M, van Krieken JH (2013). Recognizing nodal marginal zone lymphoma: recent advances and pitfalls. A systematic review. Haematologica.

[8] Arcaini L, Paulli M, Boveri E (2004). Splenic and nodal marginal zone lymphomas are indolent disorders at high hepatitis C virus seroprevalence with distinct presenting features but similar morphologic and phenotypic profiles. Cancer.

[9] Zucca E, Rossi D, Bertoni F (2023). Marginal zone lymphomas. Hematol Oncol.

[10] Kanani J, Sheikh MI (2024). An autopsy presentation of spontaneous splenic rupture in chronic myeloid leukemia: a rare case report. J Med Surg Pub Health.

